# Factors Associated With Cervical Cancer Awareness Among Women of Reproductive Age in the Democratic Republic of the Congo: Evidence From a Nationwide DHS Analysis

**DOI:** 10.1002/cnr2.70633

**Published:** 2026-08-02

**Authors:** Jovinary Adam, Olivier Mukuku, Stanislas Maseb’a Mwang Sulu, Joseph Bulanda Nsambi, Janvier Mwemedi Tawi, Alphoncina Kagaigai, Pankras Luoga

**Affiliations:** ^1^ Department of Development Studies School of Public Health and Social Sciences, Muhimbili University of Health and Allied Sciences Dar es Salaam Tanzania; ^2^ Institut Supérieur des Techniques Médicales de Lubumbashi Lubumbashi Democratic Republic of the Congo; ^3^ Centre Hospitalier Nganda de Kinshasa Kinshasa Democratic Republic of the Congo; ^4^ Department of Obstetrics and Gynaecology Faculty of Medicine, University of Lubumbashi Lubumbashi Democratic Republic of the Congo

**Keywords:** cervical cancer awareness, Democratic Republic of the Congo, Demographic and Health Survey, reproductive age, women

## Abstract

**Background:**

Cervical cancer (CC) is a major public health concern, ranked as the fourth leading cause of cancer. Evidence shows that CC is preventable if detected and treated at the early stages. In the Democratic Republic of the Congo (DRC), national‐level evidence on women’s awareness of CC and its associated factors remains limited.

**Aims:**

This study aimed to assess the level of cervical cancer awareness and identify factors associated with awareness among women of reproductive age in the DRC.

**Introduction:**

Cervical cancer (CC) is a major public health concern, ranked as the fourth leading cause of death. Evidence shows that CC is preventable if diagnosed at the early stages. In the Democratic Republic of the Congo (DRC), there is limited evidence at the national level and on factors associated with CC awareness among women of reproductive age. This study aimed to address this gap.

**Methods:**

The study analysed secondary data from a total weighted sample of 27 583 women aged 15–49, drawn from the 2023–2024 DRC Demographic and Health Survey (DRCDHS). The dependent variable was CC awareness, and the independent variables were the sociodemographic characteristics of a woman. Descriptive, bivariable and multivariable analyses were conducted. A significant factor was determined at *p*‐value < 0.05 at 95% confidence intervals (CIs).

**Results:**

The overall CC awareness among women of reproductive age in DRC was 21.3% (95% CI = 19.5–23.2). Logistic regression results revealed that women aged 25–34 (AOR = 1.36; 95% CI = 1.12–1.66) and 35–49 (AOR = 1.60; 95% CI = 1.27–2.02), women who were residing in urban areas (AOR = 1.53; 95% CI = 1.06–2.20), women with primary education (AOR = 1.45; 95% CI = 1.03–2.04), secondary education and above (AOR = 1.73; 95% CI = 1.22–2.45), employed women (AOR = 1.48; 95% CI = 1.20–1.84), women who had been examined for breast cancer (AOR = 4.72; 95% CI = 2.91–7.67), women who had been listening to radio (AOR = 1.60; 95% CI = 1.28–1.99), women who had visited health facility last 12 months (AOR = 1.63; 95% CI = 1.35–1.97), Christian (AOR = 2.35; 95% CI = 1.06–5.21), use internet (AOR = 1.67; 95% CI = 1.23, 2.28), reading newspaper/magazine (AOR = 1.33; 95% CI = 1.01–1.76) and delivered at health facility (AOR = 1.53; 95% CI = 1.18–1.98) were statistically significant associated with having CC awareness compared to their counterparts.

**Conclusion:**

The study revealed overall suboptimal CC awareness among women of reproductive age in DRC. Although health education interventions should target all women of reproductive age, special attention should be given to younger women aged 15–24 years, those with no formal education and those living in rural areas, as these subgroups demonstrated particularly lower awareness levels. Strengthening tailored interventions for these vulnerable groups may therefore improve awareness within the broader reproductive‐age population and the community at large.

**Abbreviations**
AOR: adjusted odds ratio
CC: cervical cancer
CI: confidence interval
DHS: Demographic and Health Survey
DRC: Democratic Republic of Congo
HPV: human papillomavirus
OR: odds ratio
SSA: sub‐Saharan Africa

## 1 Introduction

Cervical cancer (CC) remains a significant global health challenge despite being highly preventable. In 2022, an estimated 661 021 new cases and 348 189 deaths were recorded worldwide, making CC the fourth most common cancer and the fourth leading cause of cancer death among women [1, 2]. Without strengthened prevention strategies, the global burden is expected to rise markedly by 2050 [2]. CC disproportionately affects women in low‐ and middle‐income countries, which account for almost 90% of new cases and 85% of related deaths, reflecting persistent inequities in HPV vaccination, early detection and access to treatment [3, 4].

Sub‐Saharan Africa (SSA) bears the highest global burden of CC, with 118 013 new cases and 76 189 deaths in 2022, reflecting some of the world's highest age‐standardised incidence and mortality rates [1]. Eighteen of the 20 most affected countries are in the WHO African Region, highlighting profound regional inequities [4]. Without strengthened prevention and control efforts, incidence and mortality in SSA are projected to increase by over 50% by 2040, driven by low HPV vaccination coverage, limited and poor‐quality screening, systemic health system constraints and delays along the diagnostic–treatment pathway [3]. Although CC screening is a highly effective strategy to reduce incidence and mortality, uptake remains low in most LMICs [5, 6, 7]. Previous studies across SSA have shown that awareness of CC has been reported to be associated with screening behaviour [8, 9, 10]. However, structural barriers such as distance to health facilities, shortages of trained staff, limited availability of screening technologies and inadequate health promotion continue to impede access even where awareness is relatively high [6, 11, 12]. Limited awareness of CC has been documented in several studies in the region and may reflect broader gaps in exposure to health information, which could influence engagement with screening services [13, 14].

In the Democratic Republic of the Congo (DRC), CC represents the leading cancer affecting women. In 2022, the country recorded approximately 8705 new cases (16.5% of all female cancers) and 6187 deaths, with an estimated 5‐year prevalence of 17 175 women living with the disease [1]. Despite this considerable burden, uptake of CC screening remains suboptimal, and national‐level evidence on awareness of CC among women of reproductive age is still scarce. Most previous studies in the DRC were restricted to localised samples and lacked national representativeness, limiting understanding of the true prevalence of awareness and its associated factors [15, 16]. These studies also highlight the burden of CC with frequent late‐stage presentation and a high prevalence of high‐risk HPV genotypes (particularly HPV 16, 18, 35 and 52), contributing to poor outcomes [17].

Recent health initiatives, including the introduction of HPV vaccination in 2023 with support from WHO and GAVI, offer a major opportunity to reduce the CC burden. The program targets girls aged 9–14 years, primarily using the quadrivalent HPV vaccine (Gardasil) [18]. However, early evidence suggests suboptimal coverage in rural and peri‐urban areas due to logistical, cultural and educational barriers, underscoring the need for improved community engagement and strengthened awareness strategies [18, 19].

Awareness of CC may represent an important first step toward engagement with prevention programs, including screening and HPV vaccination [20]. However, awareness alone does not necessarily indicate adequate knowledge, positive attitudes, or participation in preventive services. Yet, nationally representative evidence on the level of CC awareness and its associated factors among women of reproductive age in the DRC remains scarce. Previous analyses with small sample sizes, limited geographic coverage, or facility‐based data do not provide sufficient insight into population‐level patterns [17, 21].

Therefore, this study aims to address this critical awareness gap by assessing national‐level CC awareness and its associated factors among women aged 15–49 years, using data from the 2023–2024 DRC Demographic and Health Survey (2023–2024 DRC DHS). Understanding the factors associated with CC awareness is essential for informing evidence‐based strategies to improve public awareness and guide the development of effective communication interventions for prevention programs.

## 2 Materials and Methods

### 2.1 Study Design

The DHS employed a two‐stage stratified cluster sampling design. In the first stage, clusters were selected within strata defined by place of residence (urban/rural), based on the 1984 General Population and Housing Census, updated using administrative and electoral data. In the second stage, households were systematically selected within each cluster, and all eligible women were interviewed.

The survey collected demographic and health‐related indicators, including socio‐demographic characteristics and CC awareness. The unit of analysis was women aged 15–49 years at the time of data collection. Detailed methodological procedures are provided in the DHS final report [22]. All analyses accounted for the complex survey design using sampling weights, primary sampling units (clusters) and stratification variables applied through the Stata *svyset* command. Descriptive, bivariable and multivariable analyses were conducted using survey‐weighted procedures to ensure correct variance estimation and nationally representative results. The 2023–2024 DHS achieved very high response rates, with 99.9% of selected households and 99% of eligible women completing the interview, thereby minimising the potential for non‐response bias.

### 2.2 Study Population

The study population included 27 583 women of reproductive age (15–49 years) who participated in the 2023–2024 DRC DHS. There were no missing responses for the outcome variable (CC awareness). For independent variables, some non‐applicable responses were identified due to DHS skip patterns (e.g., place of delivery, which was assessed only among women with a recent live birth). These responses were considered structurally non‐applicable rather than missing data and were retained as separate categories in the analysis when applicable. The final multivariable logistic regression model was estimated using the full analytical sample of 27 583 women.

Consequently, the effective analytical sample varied slightly across models depending on the variables included. The sample remains nationally representative due to the application of survey weights and the DHS complex two‐stage stratified cluster sampling design.

### 2.3 Study Variables

#### 2.3.1 Outcome Variable

The outcome of interest was CC awareness among women aged 15–49 years. Respondents were asked whether they had ever heard of CC, with responses coded as 1 for ‘Yes’ and 0 for ‘No’. There were no missing values for this variable. This single‐item measure is the standard DHS indicator for assessing population‐level awareness and has been widely used in studies across SSA.

This indicator reflects basic recognition of CC and does not capture detailed knowledge, attitudes, or preventive behaviours. Although the DHS questionnaire includes items related to screening awareness, these were not included in the analysis because they were not consistently available for all respondents and represent a distinct construct from general awareness of the disease. Accordingly, the study used ‘ever heard of cervical cancer’ as the most consistently measured and comparable indicator available in the DHS dataset.

#### 2.3.2 Exposure Variables

The independent variables consisted of three groups: (1) sociodemographic factors, including age, education level, marital status, wealth status, employment status and religion; (2) health access and utilisation, including health insurance coverage, health facility visits, current contraceptive use, place of delivery and breast cancer examination; and (3) media exposure, including listening to the radio, watching television, reading newspapers or magazines and internet use. The explanatory variables were chosen based on their availability within the data set and on the relevant literature [23, 24]. Place of delivery was assessed only among women who had a recent live birth during the DHS reference period; therefore, this variable did not apply to women without a recent birth. Examined breast cancer refers to a self‐reported history of breast examination performed by a healthcare provider and is used in this study as an indicator of contact with preventive health services rather than a measure of breast cancer knowledge. Each variable was categorised according to standard DHS coding: education level (none, primary, secondary, higher), wealth status (quintiles) and age (15–24, 25–34, 35–49). Variables were selected based on availability in the dataset and previous evidence linking them to CC awareness.

### 2.4 Statistical Analysis

The study employed Stata version 18 to perform data analyses. Descriptive analyses were conducted using weighted frequencies and percentages to estimate the prevalence of CC awareness and describe the background characteristics of the study participants. The *svyset* command was used in Stata to account for sampling weights, clustering and stratification in all analyses.

Missing values and non‐applicable responses for explanatory variables were assessed before the analysis and are reported in Table 1. The variable place of delivery was collected only among women who had a recent live birth during the DHS reference period and was therefore not applicable to women without a recent birth. These non‐applicable observations represented structural non‐applicability arising from the DHS questionnaire skip pattern rather than item non‐response. To preserve the representativeness of the study population, structurally non‐applicable responses were retained as a separate category in the multivariable analysis.

**TABLE 1:** Background characteristics of the study respondents (*N* = 27 583) (weighted sample).

Variable	Frequency (*N*)	Percentage (%)
Age (years)		
15–24	12 209	44.2
25–34	7661	27.8
35–49	7713	28.0
Place of residence		
Rural	16 177	58.7
Urban	11 406	41.4
Educational level		
No formal education	4068	14.8
Primary	6488	23.5
Secondary and above	17 027	61.7
Wealth status		
Poor	10 049	36.4
Middle	5134	18.6
Rich	12 400	45.0
Employment status		
Unemployed	11 001	39.9
Employed	16 582	60.1
Marital status		
Never in union	9418	34.1
Married	15 683	56.9
Separated	2482	9.0
Covered by health insurance		
No	26 433	95.8
Yes	1150	4.2
Current contraceptive use		
No	24 854	90.1
Yes	2729	9.9
Listening to the radio		
No	18 908	68.6
Yes	8675	31.5
Watching television		
No	19 183	69.6
Yes	8400	30.5
Visited health facility in the last 12 months		
No	19 274	69.9
Yes	8309	30.1
Examined breast cancer		
No	26 952	97.7
Yes	631	2.3
Religion		
Non‐religion	287	1.0
Traditional	818	3.0
Islam	402	1.5
Christianity	26 076	94.5
Use of internet		
No	22 420	81.3
Yes	5163	18.7
Reading a newspaper or a magazine		
No	24 156	87.6
Yes	3427	12.4
Place of delivery^a^		
Home/elsewhere	1876	6.8
Health facility	9068	32.9
Not applicable	16 639	60.3

^a^ Place of delivery was assessed only among women who had a recent live birth during the DHS reference period (*n* = 10 944). Women who were not eligible for this question were retained as a ‘not applicable’ category, reflecting the DHS skip pattern rather than missing data.

Multicollinearity among independent variables was assessed using the variance inflation factor (VIF). All VIF values were below the commonly accepted threshold of 10, with the highest being 3.7, indicating no evidence of problematic multicollinearity. The chi‐square test was used to assess associations between CC awareness and categorical independent variables. Variables with a *p*‐value < 0.25 in the bivariable analyses were included in the multivariable logistic regression model.

A multivariable logistic regression model was fitted to identify factors independently associated with CC awareness. The final multivariable model included all study participants (*N* = 27 583). Variables with *p*‐values < 0.05 in the multivariable model were considered statistically significant. The strength of association was expressed using adjusted odds ratios (AORs) and their corresponding 95% confidence intervals (CIs).

### 2.5 Ethical Considerations

The 2023–2024 DRC DHS was conducted with ethical approval from the relevant national and international institutional review boards before data collection. Written informed consent was obtained from all participants before data collection. For respondents aged 15–17 years, consent procedures were implemented in accordance with DHS ethical guidelines and national regulations.

This study was based on a secondary analysis of fully anonymised DHS data accessed through the DHS Program following approval of a formal data request process. The dataset contains no personal identifiers, thereby ensuring participant confidentiality and privacy. Permission to access and use the data for this research was obtained from the DHS Program through a formal data request process. Because this study involved analysis of fully de‐identified secondary data and did not involve any interaction with participants or access to identifiable information, no additional ethical approval was required from the authors' institutions, in accordance with the national guidelines governing research involving anonymised secondary data in the DRC [25].

## 3 Results

### 3.1 Background Characteristics of the Study Respondents

A total sample of 27 583 women of reproductive age (15–49 years) was included in the analysis for this study. The largest proportion of respondents was aged 15–24 years (12 209; 44.2%). More than half of the participants resided in rural areas (16 177; 58.7%). More than half, 17 027 (61.7%), of the study respondents had secondary education and above, while 4068 (14.8%) reported having no education. Nearly half, 12 400 (45%), were classified in the rich wealth quintile and the majority, 24 854 (90.1%), were not covered by health insurance. More than half, 16 582 (60.1%), reported that they were employed. The characteristics of the respondents are presented in Table 1.

### 3.2 Bivariate Analysis Between Cervical Cancer Awareness and Independent Variables Among Women of Reproductive Age in the DRC

The results from the bivariate analysis indicated that the percentage of CC awareness increases with age, rising from 16.5% among women aged 15–24 years to 25.8% among those aged 35–49 years. Women residing in urban areas had a higher proportion (29.7%) of CC awareness compared to those in rural areas. Additionally, CC awareness increases with education level, from 11.1% among those with no education to 25.7% among those with secondary education and above. The percentage of CC awareness is higher among women from wealthy households (29%) compared to those from poor households. Moreover, employed women had a higher percentage of CC awareness (24.3%) compared to unemployed women, as shown in Table 2.

**TABLE 2:** Proportions of cervical cancer awareness across independent variables (bivariate analysis).

Variable	Number of women with awareness of cervical cancer	Percentage (%)	95% confidence intervals	*p*
Age (years)				**< 0.001**
15–24	2020	16.5	[14.80, 18.46]	
25–34	1857	24.2	[21.83, 26.81]	
35–49	1993	25.8	[23.51, 28.32]	
Place of residence				**< 0.001**
Rural	2481	15.3	[13.45, 17.44]	
Urban	3389	29.7	[26.64, 32.98]	
Educational level				**< 0.001**
No education	453	11.1	[8.846, 13.93]	
Primary	1041	16	[14.22, 18.05]	
Secondary and above	4376	25.7	[23.43, 28.11]	
Wealth status				**< 0.001**
Poor	1442	14.4	[12.70, 16.18]	
Middle	808	15.7	[13.49, 18.26]	
Rich	3620	29.2	[26.24, 32.34]	
Employment status				**< 0.001**
Unemployed	1845	16.8	[14.72, 19.04]	
Employed	4025	24.3	[22.20, 26.47]	
Marital status				**< 0.001**
Never in union	1814	19.3	[17.03, 21.71]	
Married	3447	22	[20.10, 23.98]	
Separated	609	24.5	[21.62, 27.72]	
Covered by health insurance				**< 0.001**
No	5352	20.2	[18.58, 22.03]	
Yes	518	45	[37.87, 52.39]	
Current contraceptive use				**< 0.001**
No	5026	20.2	[18.35, 22.24]	
Yes	844	30.9	[27.18, 34.96]	
Listening to the radio				**< 0.001**
No	3275	17.3	[15.74, 19.02]	
Yes	2595	29.9	[27.18, 32.79]	
Watching television				**< 0.001**
No	3328	17.3	[15.73, 19.09]	
Yes	2542	30.3	[26.92, 33.83]	
Visited a health facility last 12 months				**< 0.001**
No	3472	18	[16.26, 19.92]	
Yes	2398	28.9	[26.21, 31.66]	
Examined breast cancer				**< 0.001**
No	5421	20.1	[18.42, 21.92]	
Yes	449	71.1	[63.15, 77.90]	
Religion				**0.042**
Non‐religion	26	9	[4.811, 16.35]	
Traditional	150	18.4	[12.53, 26.14]	
Islam	67	16.6	[10.29, 25.54]	
Christianity	5627	21.6	[19.74, 23.54]	
Use of the internet				**< 0.001**
No	3871	17.3	[15.86, 18.77]	
Yes	1999	38.7	[34.64, 42.98]	
Reading a newspaper or a magazine				**< 0.001**
No	4648	19.2	[17.53, 21.08]	
Yes	1222	35.7	[31.98, 39.50]	
Place of delivery				**< 0.001**
Home/elsewhere	217	11.5	[9.188, 14.41]	
Health facility	1983	21.9	[19.68, 24.22]	
Not applicable	3675	22.1	[21.5, 22.7]	

*Note:* Bold values indicate statistical significance *p* < 0.05.

### 3.3 Cervical Cancer Awareness Among Women of Reproductive Age in DRC

**FIGURE 1:**
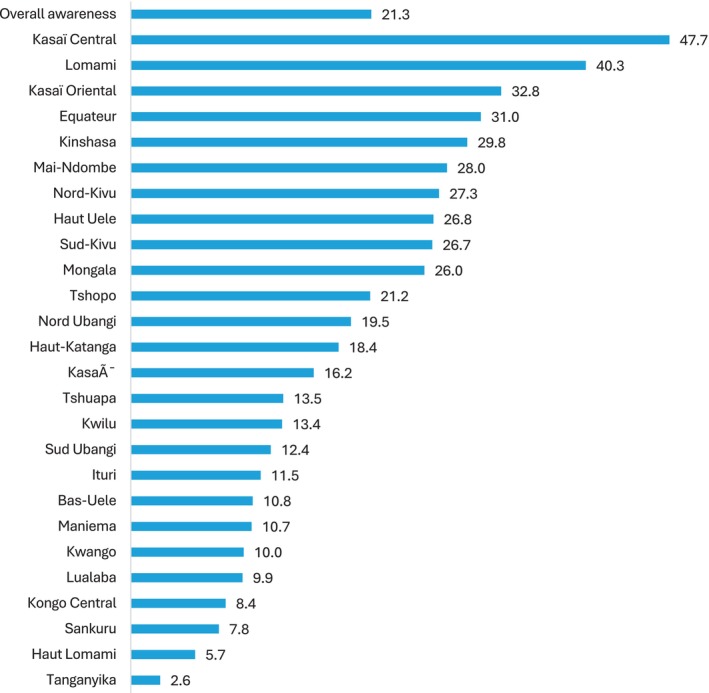
Cervical cancer awareness among women of reproductive age across provinces in the DRC (%).

The study found that overall awareness of CC among women of reproductive age in the DRC was 21.3% (95% CI = 19.5, 23.2). This varied across provinces in the DRC, ranging from 2.6% in Tanganyika to 47.7% in Kasaï Central, as indicated in Figure 1.

### 3.4 Factors Associated With Cervical Cancer Awareness Among Women of Reproductive Age in DRC

The logistic regression results revealed that women aged 25–34 (AOR = 1.36; 95% CI = 1.12–1.66) and 35–49 (AOR = 1.60; 95% CI = 1.27–2.02) had higher odds of CC awareness compared to those aged 15–24 years. Women residing in urban areas (AOR = 1.53; 95% CI = 1.06–2.20), employed women (AOR = 1.48; 95% CI = 1.20–1.84), and those who had been examined for breast cancer (AOR = 4.72; 95% CI = 2.91–7.67) had higher odds of CC awareness compared to their counterparts. The odds of CC awareness were higher among women with primary education (AOR = 1.45; 95% CI = 1.03–2.04), secondary education and above (AOR = 1.73; 95% CI = 1.22–2.45) compared to women with no education. Moreover, women who had listening to radio (AOR = 1.60; 95% CI = 1.28–1.99), visited health facility last 12 months (AOR = 1.63; 95% CI = 1.35–1.97), those who are Christian (AOR = 2.35; 95% CI = 1.06–5.21), use internet (AOR = 1.67; 95% CI = 1.23–2.28), reading newspaper/magazine (AOR = 1.33; 95% CI = 1.01–1.76) and delivered at health facility (AOR = 1.53; 95% CI = 1.18–1.98), were significantly associated with CC awareness among women of reproductive age in DRC as presented in Table 3.

**TABLE 3:** Crude and adjusted odds ratios for factors associated with cervical cancer awareness among women of reproductive age in the Democratic Republic of the Congo.

Variable	Crude odds ratio	95% confidence interval	*p*	Adjusted odds ratio	95% confidence interval	*p*
Age (years)						
15–24	1.00			1.00		
25–34	1.61	1.43–1.82	< 0.001	1.36	1.12–1.66	0.002
35–49	1.76	1.57–1.96	< 0.001	1.60	1.27–2.02	< 0.001
Place of residence						
Rural	1.00			1.00		
Urban	2.33	1.88–2.90	< 0.001	1.53	1.06–2.20	0.022
Educational level						
No education	1.00			1.00		
Primary	1.52	1.17–1.98	0.002	1.45	1.03–2.04	0.035
Secondary and above	2.76	2.12–3.59	< 0.001	1.73	1.22–2.45	0.002
Wealth status						
Poor	1.00			1.00		
Middle	1.11	0.91–1.36	0.294	0.84	0.66–1.06	0.142
Rich	2.46	2.01–3.02	< 0.001	0.92	0.63–1.35	0.676
Employment status						
Unemployed	1.00			1.00		
Employed	1.59	1.39–1.83	< 0.001	1.48	1.20–1.84	< 0.001
Marital status						
Never in union	1.00			1.00		
Married	1.18	1.05–1.33	0.007	1.35	0.96–1.88	0.085
Separated	1.36	1.15–1.62	< 0.001	1.26	0.83–1.92	0.271
Covered by health insurance						
No	1.00			1.00		
Yes	3.23	2.41–4.31	< 0.001	1.45	0.98–2.14	0.061
Current contraceptive use						
No	1.00			1.00		
Yes	1.77	1.43–2.18	< 0.001	1.14	0.87–1.49	0.346
Listening to the radio						
No	1.00			1.00		
Yes	2.04	1.80–2.30	< 0.001	1.60	1.28–1.99	< 0.001
Watching television						
No	1.00			1.00		
Yes	2.07	1.72–2.48	< 0.001	1.72	0.54–2.98	0.120
Visited a health facility last 12 months						
No	1.00			1.00		
Yes	1.85	1.62–2.10	< 0.001	1.63	1.35–1.97	< 0.001
Examined breast cancer						
No	1.00			1.00		
Yes	9.76	6.90–13.79	< 0.001	4.72	2.91–7.67	< 0.001
Religion						
Non‐religion	1.00			1.00		
Traditional	2.27	1.02–5.04	0.045	2.21	0.86–5.69	0.101
Islam	2.00	0.83–4.78	0.121	1.10	0.41–3.01	0.846
Christianity	2.77	1.42–5.41	0.003	2.35	1.06–5.21	0.035
Use of the internet						
No	1.00			1.00		
Yes	3.03	2.54–3.62	< 0.001	1.67	1.23–2.28	< 0.001
Reading a newspaper or a magazine						
No	1.00			1.00		
Yes	2.33	1.97–2.74	< 0.001	1.33	1.01–1.76	0.039
Place of delivery						
Home/elsewhere	1.00			1.00		
Health facility	2.14	1.64–2.80	< 0.001	1.53	1.18–1.98	< 0.001
Not applicable	2.17	1.87–2.51	< 0.001	1.60	1.20–2.02	< 0.001

*Note:* The multivariable logistic regression model included all women of reproductive age (*N* = 27 583). The place‐of‐delivery variable was assessed only among women who had a recent live birth during the DHS reference period (*n* = 10 944). Women who were not eligible for this question were retained as a ‘not applicable’ category, reflecting the DHS skip pattern rather than missing data.

## 4 Discussion

To our knowledge, this study provides the first nationally representative evidence on CC awareness among women of reproductive age in the DRC. The overall rate of awareness was only 21.3%, which is substantially lower than the rates reported in many SSA countries. Multivariable analysis revealed that socio‐demographic characteristics (age, urban residence, education, employment), health system factors (recent facility visits, health facility as place of delivery, breast cancer examination) and media exposure (radio, internet, newspapers) were the most consistent factors associated with awareness. Conversely, wealth index, health insurance coverage, marital status and contraceptive use did not retain statistical significance after adjustment. These findings highlight a multifaceted set of factors associated with CC awareness that need to be considered for effective CC awareness strategies in the DRC and for informing health communication strategies.

Compared to other studies conducted in SSA, our study reports a proportion of awareness of only 21.3%, which remains low in comparison with findings from other countries in the region. For instance, CC awareness has been reported at 11% in Benin, 36% in Mozambique [26], 37.5% in Nigeria [27], 38% in Madagascar, 42.2% in Ethiopia [28], 49% in Mauritania and 53% in Cameroon [26], 72% in Botswana [29] and 78%–80% in Kenya [14, 30]. These figures illustrate wide disparities both within and between countries. This pronounced disparity highlights the urgent need for sustained investment in health promotion in the DRC, where CC remains the leading cause of cancer‐related mortality among women [18]. Low awareness may contribute to limited engagement with CC prevention programmes. Previous studies in the DRC have documented low screening uptake and frequent late‐stage presentation of CC in hospital settings [31]. These findings highlight the critical need for context‐specific educational interventions and community‐based awareness strategies to enhance early detection and reduce CC morbidity and mortality in the DRC. Several studies have shown that women's knowledge of CC strongly influences their participation in screening programs [29, 32, 33, 34] and low awareness remains a key barrier to effective CC prevention.

Age was an important factor associated with CC awareness in our study. Women aged 25–34 and 35–49 years had significantly higher awareness than those aged 15–24 years. Older women are more likely to be aware and have been screened for CC, as they tend to have greater exposure to reproductive health services and health education through pregnancy, childbirth and repeated facility visits. These encounters create multiple opportunities for counselling and access to screening, as demonstrated in a recent multi‐country analysis from SSA [35] as well as studies conducted in specific countries [24]. In contrast, adolescents and young women remain particularly vulnerable due to early sexual debut and a higher risk of HPV infection, the primary etiological factor for CC [36]. Evidence from Kenya further highlights that while adolescents often report high rates of sexual initiation, their limited knowledge significantly influences the acceptability and uptake of prevention strategies [37]. Taken together, these findings underscore the need to expand adolescent‐friendly health services, strengthen school‐based education and design‐targeted community interventions that reach younger women early, thereby improving exposure to CC information and promoting health awareness at an early stage.

Place of residence was significantly associated with CC awareness, with women in urban areas consistently demonstrating higher awareness than their rural counterparts. These urban–rural disparities likely reflect differences in access to health facilities, exposure to mass media, literacy and overall health system infrastructure. In the DRC, where more than 60% of the population lives in rural areas, this gap represents a substantial equity challenge. Similar urban–rural patterns have been observed across SSA, where women in urban settings are more frequently exposed to CC information and have higher levels of screening utilisation [38, 39]. Addressing these inequities requires context‐adapted strategies, including mobile outreach programs, integration of CC education and screening into primary care and engagement of community health workers. Evidence from the region demonstrates the effectiveness of such interventions: mobile HPV self‐sampling in Uganda, community health worker‐led recruitment in Cameroon [40], integration of screening into routine primary care in West Africa [41], home‐based educational initiatives in Uganda [42] and integration of screening into reproductive health services in South Africa [43], all enhance exposure to CC information among underserved populations.

Literacy emerged as one of the strongest factors associated with CC awareness, with women who had attained primary or secondary and higher education reporting significantly greater awareness compared to those without formal education. Higher educational attainment generally facilitates access to health information, comprehension of preventive messages and autonomy in health decision‐making. In addition, targeted health education interventions have been shown to increase awareness among African women, particularly when delivered through peer health educators and culturally tailored approaches, some of which have also improved uptake of CC screening [44]. By contrast, findings from Benin and Mozambique suggest that literacy alone does not always predict awareness, as cultural or systemic barriers may limit the benefits of formal schooling [26]. The persistently low levels of awareness among uneducated women in the DRC emphasise the need for alternative strategies, such as simplified, pictorial communication materials and community‐based interventions, to ensure that women with limited literacy are not left behind.

Beyond literacy, healthcare utilisation is a factor associated with CC awareness. Women who recently visited a health facility or delivered at one were more likely to be informed, highlighting routine consultations and childbirth‐related care as key opportunities for exposure to cancer prevention information. Nevertheless, despite 30.1% of Congolese women reporting a recent facility visit and 82.8% delivering in health facilities, overall awareness remains low, indicating these contacts are underutilised. As Olivieri et al. [26] and Chepkorir et al. [45] noted, healthcare providers are trusted sources whose engagement can enhance knowledge, literacy, attitudes and screening practices. Still, cross‐country data reveal missed opportunities when education is not consistently integrated into service delivery. Embedding CC counselling into antenatal, delivery, family planning and postnatal services in the DRC, therefore, represents a critical pathway to expand awareness effectively. These findings align with WHO's Cervical Cancer Awareness Month 2024 campaign [46], emphasising routine education, HPV vaccination and screening, particularly for women with limited access and low literacy.

Another striking observation is the strong association between breast cancer examination and CC awareness, with women who have ever undergone breast examination being nearly five times more likely to report awareness. This association is interpreted as reflecting exposure to healthcare services and integrated preventive health messaging rather than direct knowledge of breast cancer. This finding reflects the potential synergies of integrated cancer prevention programs, where contact with one cancer‐related intervention increases exposure to information about others [25, 47]. Cross‐country evidence also suggests that such synergies are not consistently exploited; for instance, in Benin and Mozambique, limited integration across cancer programs has constrained awareness gains [25]. By contrast, integrated strategies tested in community‐based models, including lay health advisors and group education sessions, have been shown to improve knowledge and uptake of both breast and CC screening [47].

Although health insurance coverage was associated with CC awareness in bivariate analyses, this association lost significance in adjusted models, likely due to the extremely low coverage in the DRC, estimated at less than 5% [48]. This finding aligns with previous reports indicating inconsistent associations between socioeconomic indicators and awareness. For instance, Olivieri et al. found that health insurance and other structural markers, such as wealth index, were not consistently associated with CC awareness across multiple countries, including Benin, Mauritania and Mozambique [26]. Conversely, studies in other resource‐constrained settings have reported a positive association between wealth, education and knowledge or utilisation of screening services [49]. Taken together, these patterns suggest that structural socioeconomic indicators, including wealth and education, may not reliably predict CC awareness in all African contexts. Public health programs should therefore prioritise targeted, individualised strategies that leverage direct service contacts and community engagement. Evidence suggests that integrating CC education into primary care, antenatal and community‐based services through lay health advisors, discussion groups and local media may improve women's exposure to CC information [50, 51]. Strengthening universal health coverage may indirectly increase opportunities for awareness, but embedding health education at every health system contact remains a feasible approach in the Congolese context [52] and supports targeted information campaigns.

In addition, exposure to media and information constitutes an increasingly important avenue for health education and community sensitisation. Radio and microphone announcements, in particular, remain the most accessible and widely used source of health information [26, 53], making it a highly effective channel for targeted campaigns in local languages. Television, although less consistently available in rural settings, was associated with higher awareness in Mauritania and Mozambique, indicating its complementary role in reinforcing health messages [26]. Similarly, internet use and mobile phone ownership were linked to greater awareness across several countries, reflecting the growing influence of digital platforms in health literacy despite limited penetration in SSA [26]. Print media, including newspapers and magazines, were also associated with higher awareness among literate urban populations, confirming their continued relevance [53]. Taken together, these findings highlight the need for multi‐channel, context‐specific strategies that combine traditional media, digital platforms, mobile technologies and community‐based interventions, while accounting for literacy and healthcare decision‐making dynamics, to maximise outreach and reduce information inequities, particularly among rural and underserved populations in the DRC.

Religious affiliation significantly shapes CC awareness in the DRC. Christian women generally show higher levels of CC awareness, which may partly reflect the role of faith‐based organisations, which manage over 40% of health facilities and leverage trusted community leaders to promote health education. Conversely, certain religious norms may influence women's exposure to health information and their interaction with health services. Among Muslim women, modesty concerns and preferences for providers of the same gender or faith may affect engagement with health services, although evidence on these associations remains context‐specific [54]. These findings suggest that religion may shape pathways of information dissemination and access to health communication channels. Faith‐based networks, therefore, represent important platforms for culturally appropriate health education interventions in the DRC [55].

The findings highlight several actionable strategies. First, CC education should be systematically integrated into routine health service contacts, particularly during healthcare facility visits and institutional delivery services. Second, community media should be leveraged, with radio in local languages and mobile‐based communication prioritised for rural populations. Third, school‐ and adolescent‐focused interventions are essential to improve awareness among young women. Fourth, faith‐based organisations should be engaged as key partners in awareness‐raising and health communication. Fifth, integrated cancer education strategies combining breast and CC information can maximise resources and outreach. Finally, equity gaps must be addressed through targeted health communication strategies for uneducated, rural and unemployed women who remain the most disadvantaged in terms of exposure to CC information.

### 4.1 Strengths and Limitations of the Study

This study used a large nationally representative DHS dataset and incorporated survey weights, clustering and stratification, thereby enhancing the generalisability of the findings to women of reproductive age in the DRC. The use of a standardised DHS questionnaire also facilitates comparisons with studies conducted in other low‐ and middle‐income countries.

However, several limitations should be considered. First, the cross‐sectional design precludes any causal inference between the factors examined and CC awareness. Second, the outcome variable was based on a single DHS question (‘ever heard of cervical cancer’), which captures only basic awareness rather than comprehensive knowledge of CC, including understanding of symptoms, risk factors and prevention methods. Therefore, the study identifies population groups with lower exposure to information about CC, rather than groups with poor knowledge or preventive behaviours. Third, although survey weights were applied to account for unequal probabilities of selection, clustering and stratification, they do not fully correct for potential non‐response bias. If non‐response was differential—particularly among women with lower education, rural residence, or limited access to media—it may have introduced non‐response bias, potentially resulting in either overestimation or underestimation of CC awareness. Although response rates were very high, residual non‐response bias cannot be entirely excluded. Fourth, the self‐reported nature of the data may be subject to recall and social desirability biases. Fifth, the study population (women aged 15–49 years) reflects the DHS sampling framework and does not directly correspond to CC prevention target groups. HPV vaccination programmes primarily target adolescent girls aged 9–14 years, while CC screening is generally recommended for women aged 30–49 years. Therefore, the findings should not be interpreted as evidence of screening programme readiness or vaccination targeting, but rather as an assessment of CC awareness in the general reproductive‐age population.

Finally, important dimensions such as depth of knowledge, attitudes toward CC screening, HPV vaccination awareness and actual screening uptake were not available in the DHS dataset and could not be assessed.

## 5 Conclusion

The study highlighted the considerably low CC awareness among women of reproductive age in the DRC. Its positively associated factors included age, urban residence, education, employment, recent facility visits, health facility as place of delivery, breast cancer examination and media exposure. These findings highlight a multifaceted set of factors associated with CC awareness that should inform the design of effective awareness strategies in the DRC.

The government should strengthen communication and awareness‐raising interventions targeting younger women (15–24 years), women with no formal education and those living in rural areas, as these groups reported lower levels of CC awareness. These interventions should prioritise community‐based approaches through trained health workers and local leaders, integration of CC messages into reproductive and maternal health services and the use of literacy‐adapted media such as local radio or pictorial materials. Mobile outreach activities could help reach remote populations.

Future research should identify effective and context‐appropriate strategies to improve CC awareness across the DRC, including implementation and cost‐effectiveness studies in diverse geographic and resource‐limited settings.

## Author Contributions

**Stanislas Maseb’a Mwang Sulu:** writing – review and editing, validation, visualization, supervision. **Olivier Mukuku:** conceptualization, writing – original draft, writing – review and editing, visualization, methodology. **Alphoncina Kagaigai:** writing – review and editing, visualization, methodology, investigation. **Joseph Bulanda Nsambi:** visualization, writing – original draft, supervision, resources, validation. **Pankras Luoga:** conceptualization, methodology, formal analysis, software, data curation, writing – review and editing, validation. **Jovinary Adam:** conceptualization, methodology, software, data curation, formal analysis, writing – original draft, validation. **Janvier Mwemedi Tawi:** writing – original draft, software, methodology, data curation.

## Funding

The authors have nothing to report.

## Ethics Statement

DHS surveys are conducted in accordance with the ethical principles of the Declaration of Helsinki and receive ethical approval from relevant institutional review boards. This study analysed data from the third Demographic and Health Survey of the Democratic Republic of the Congo (DHS‐DRC III 2023–2024), which had obtained ethical approval before data collection. As the present study involved only a secondary analysis of de‐identified DHS data, without direct interaction with participants or access to identifiable information, no additional ethical clearance was required. Permission to access and use the dataset was obtained from the DHS Program through a formal data request process.

## Conflicts of Interest

The authors declare no conflicts of interest.

## Data Availability

The following information was supplied regarding data availability: Demographic and Health Survey EDS‐RDC III 2023–2024: https://www.dhsprogram.com.
